# Computational design of molecules for an all-quinone redox flow battery†
†Electronic supplementary information (ESI) available: The list of computationally predicted candidate quinone molecules with interesting redox properties. See DOI: 10.1039/c4sc03030c



**DOI:** 10.1039/c4sc03030c

**Published:** 2014-11-21

**Authors:** Süleyman Er, Changwon Suh, Michael P. Marshak, Alán Aspuru-Guzik

**Affiliations:** a Department of Chemistry and Chemical Biology , Harvard University , 12 Oxford Street , Cambridge , MA 02138 , USA . Email: aspuru@chemistry.harvard.edu; b Leiden Institute of Chemistry , Gorlaeus Laboratories , Leiden University , P.O. Box 9502 , 2300 RA Leiden , The Netherlands

## Abstract

Inspired by the electron transfer properties of quinones in biological systems, we recently showed that quinones are also very promising electroactive materials for stationary energy storage applications. Due to the practically infinite chemical space of organic molecules, the discovery of additional quinones or other redox-active organic molecules for energy storage applications is an open field of inquiry. Here, we introduce a high-throughput computational screening approach that we applied to an accelerated study of a total of 1710 quinone (Q) and hydroquinone (QH_2_) (*i.e.*, two-electron two-proton) redox couples. We identified the promising candidates for both the negative and positive sides of organic-based aqueous flow batteries, thus enabling an all-quinone battery. To further aid the development of additional interesting electroactive small molecules we also provide emerging quantitative structure-property relationships.

## Abstract 2

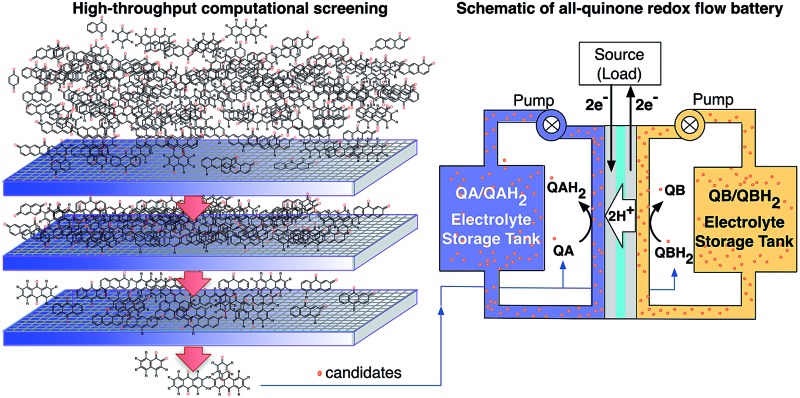
We demonstrate a successful high-throughput screening approach for the discovery of inexpensive, redox-active quinone molecules for organic-based aqueous flow batteries.

## Introduction

We recently demonstrated that quinones can be used as electroactive materials for stationary energy storage applications by creating an aqueous flow battery (AFB) based on the redox chemistry of the 9,10-anthraquinone-2,7-disulphonic acid (AQDS) and the Br_2_/Br^–^ couple.^1^ Replacing expensive redox-active metals in AFBs with abundant, carbon-based molecules, such as quinones, can dramatically lower the cost of electricity storage, because they can potentially be sourced from petroleum or biological sources.^1^ In contrast with many aqueous inorganic redox couples, such as M^3+/2+^ (M = V, Cr, or Fe), quinones undergo two-electron redox events in aqueous solution, which can enable quinone-based flow batteries to achieve higher energy densities than the conventional metal-based flow batteries.^2^ Several previous studies have computationally investigated one-electron redox couples of quinones^3–5^ yet none of trials were systematically screened for the two-electron two-proton reduction potentials that would occur in the acid electrolyte of an AFB. At present, the compiled data of the experimental reduction potentials of 700 one-electron quinone couples is the most detailed source of information.^6^ In addition, the experimental aqueous solubility data of quinones is limited to benzoquinone, hydroquinone, and some –SO_3_H functionalized quinones.^7–9^

The central aim of the present study is to systematically study the redox properties of a library of existing and non-existing quinone and hydroquinone derivatives and to investigate their quantitative structure–property relationships (QSPRs). Here, we use a virtual screening approach^10–16^ coupled with materials genomic concepts^17–19^ to allow for the rational design of an all-quinone flow battery.

Quinone–bromide flow batteries have been shown to reduce the cost of electrical energy storage by nearly an order of magnitude,^1^ and continued improvements in cell voltage and energy density could continue to drive the energy and power cost components down.^20^ Replacement of bromine with a quinone for the positive electrolyte would enable the use of low-cost storage tank, plumbing, and membrane materials.

## Methodology

### The combinatorial library of quinones/hydroquinones

**Fig. 1:**
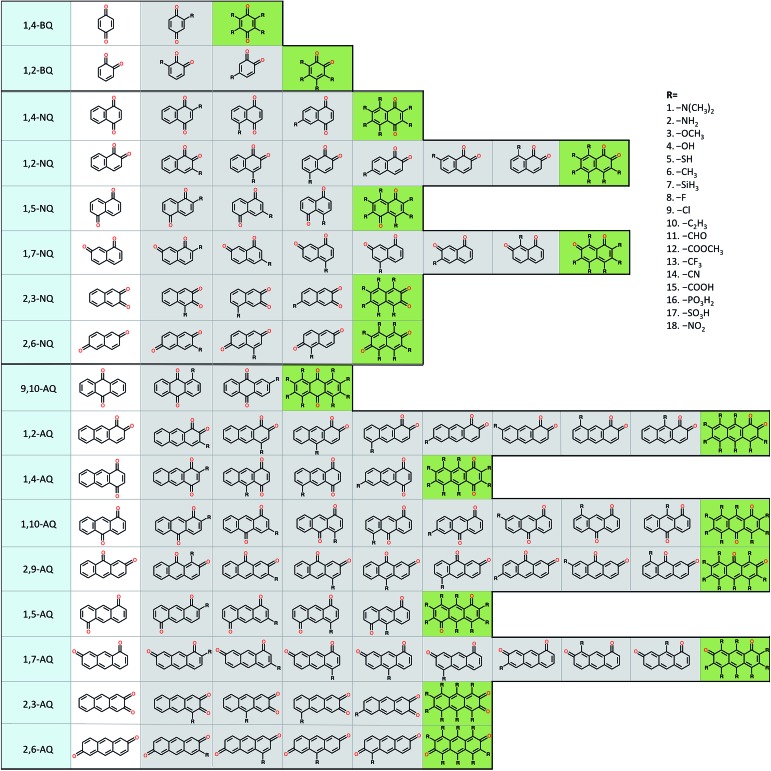
A schematic representation of the molecular screening library. The parent BQ, NQ, and AQ isomers are shown on left (white). These quinone isomers are functionalized with 18 different R-groups singly (gray) and fully (green) to generate a total of 1710 quinone molecules.

To explore the electrochemical properties of the candidate quinones we generated a virtual library of molecules by altering the core structures of quinones and by decorating quinone cores with interesting chemical substituents. The classes of 1-, 2-, and 3-ring quinone isomers are enumerated according to the position of the ketone groups on the pure quinones. The screening library also covers multiple ring quinone isomers that have the two cyclic ketone groups on different rings. Thus, the combinatorial library of the pure quinones contains 2, 6, and 9 different classes for benzoquinones (BQs), naphthoquinones (NQs), and anthraquinones (AQs), respectively (Fig. 1).

We utilized the following R-groups as substituents, –N(CH_3_)_2_, –NH_2_, –OCH_3_, –OH, –SH, –CH_3_, –SiH_3_, –F, –Cl, –C_2_H_3_, –CHO, –COOCH_3_, –CF_3_, –CN, –COOH, –PO_3_H_2_, –SO_3_H, and –NO_2_. The position of substituents is known to affect the electrochemistry of quinones significantly.^21–24^ The effects of the incorporated R-groups on the redox potential (*E* ^0^) of the quinone couples are investigated in two extreme conditions, *i.e.*, the single and the full substitutions, since *E* ^0^ and solvation free energy (Δ*G*0solv) of the intermediately substituted quinones would simply assumed to fall in between these two ends. For single substitutions, we systematically studied all possible substitution sites on a quinone. In the full substitution studies, all hydrogens of a quinone core are substituted with the same R-group. The combinatorial quinone library enumerates a total of 1710 quinone (Q)/hydroquinone (QH_2_) molecular couples.

### High-throughput computational screening

The spatial positioning of the substituted (R) groups that enable new intramolecular hydrogen bonds is known to dramatically influence the calculated total free energies of the molecules.^1,25^ To accurately predict the ground state of a molecule, such as a quinone functionalized with sterically crowded and rotatable R-groups, it is useful to study many different conformers with quantum chemical methods. Taking into account of a trade-off between number of conformers (accuracy) and available computing power (resources), for our computational screening we generated a minimum of three low-energy conformers for each of the Q (oxidized) and the QH_2_ (reduced) molecules using a generic force field.^26,27^ Thus, the screening library contains more than 10000 virtual quinone derivatives in their Q and QH_2_ three-dimensional gas phase forms. The structural optimization of all of the conformers was carried out within the framework of the density functional theory (DFT). The DFT total energies of the fully optimized molecules are used to predict the *E* ^0^ of the Q/QH_2_ couples. As a descriptor of solubility in aqueous media, we also utilized the optimized structures of the quinones to calculate the Δ*G*0solv. The entire high-throughput (HT) computational screening approach comprised approximately 33000 DFT simulations.

The *E* ^0^ of all of the Q/QH_2_ couples existing in the virtual library are predicted using the theoretical approach of the previous study.^1^ It is known that at low pHs, the reduction of a quinone to its hydroquinone requires two electrons and two protons.^24^ In a recent study, we confirmed the 2e^–^/2H^+^ process for the reduction of chemically functionalized 9,10-anthraquinones to 9,10-anthrahydroquinones.^1^ Accordingly, here, we assume that the reduction of a quinone is a single-step reaction involving a two-electron two-proton process.

Using *E* ^0^ = –(*nF*)^–1^Δ*H*_f_ + *b*, where *n* is the number of electrons, *F* is the Faraday constant, and *b* is a constant, we directly correlate the experimentally measured *E* ^0^ of Q/QH_2_ couples in aqueous solutions to that of the heat of formation of hydroquinones at 0 K, Δ*H*_f_, from the quinones and the hydrogen gas.^28^ The calculation of Δ*H*_f_ is based on the following reaction, where the two electrons and the two protons are replaced by a hydrogen molecule.

1 Q + H_2_ → QH_2_

At *T* = 0 K, the calculation of Δ*H*_f_ requires DFT total energies of quinones in their oxidized and reduced gas phase forms as well as the total energy of a hydrogen molecule. Because the entropies of reduction of quinones are found to be very similar, we neglected the entropy contributions on the free energies.^28^ As a training set for our calibration model, we utilized the experimental data on aqueous redox Q/QH_2_ couples^29^ and their DFT computed energies. ESI, Fig. S1† shows the robustness of the calibration model in predicting the *E* ^0^ for a diverse set of quinones in different chemical solvents. The developed calibration model, based on the linear correlation (*R* ^2^ = 0.974)^1^ between the calculated Δ*H*_f_ and the measured *E*0(exp), provides an accelerated way to predict the *E*0(theo) for the 1710 Q/QH_2_ couples in the virtual library of quinones. A total of 10308 quinone and hydroquinone conformers were used as input structures for the DFT geometry optimizations, and the conformers with the lowest DFT total energies are then used to predict their Δ*H*_f_, which in turn are used to estimate *E* ^0^ and Δ*G*0solv.

DFT calculations were carried out using the Perdew–Burke–Ernzerhof (PBE) functional of the generalized gradient approximation (GGA),^30^ the projector augmented wave (PAW) method,^31,32^ a plane-wave basis set, and the conjugate gradient (CG) algorithm, as implemented in the Vienna *Ab initio* Simulation Package (VASP).^33,34^ A cubic box of 25 Å along with the Γ-point sampling was used. The plane-wave kinetic energy cut-off was set at 500 eV. The convergence was assumed to be reached when the total remaining forces on the atoms were less than 10^–2^ eV Å^–1^.

In support of future synthesis efforts, it is desirable to identify the water soluble quinones. However, solubility is a complex function of crystal packing energy described by enthalpy of sublimation, cavitation energy, and solvation energy for interactions between solvent and solute.^35^ A major challenge is the determination of sublimation energy which is an arduous task due to the lack of well-defined structures and highly accurate periodic calculations.^36^ Palmer *et al.*'s work illustrates the difficulty in predicting solubility, even when using high-level theoretical calculations.^37^

In the current HT study, we treat the Δ*G*0solv as a descriptor of solubility in aqueous media. The Δ*G*0solv of the newly generated compounds is calculated by using the DFT ground state wave functions (PBE/6-31G**) and a Poisson–Boltzmann solvation model.^38–40^ This offers a compromise between the speed and the accuracy for our calculations. The Δ*G*0solv of a compound is the difference between the total energy of the solvated form and the total energy of the gas phase form. Thus, a negative value of Δ*G*0solv relates to a quinone with good aqueous solubility. We calculated Δ*G*0solv only for the oxidized form of quinones, since they would expected to be less soluble than their respective hydroquinones in water.

## Results and discussion

### Distributions of *E* ^0^ and Δ*G*0solv of the screened virtual library

**Fig. 2:**
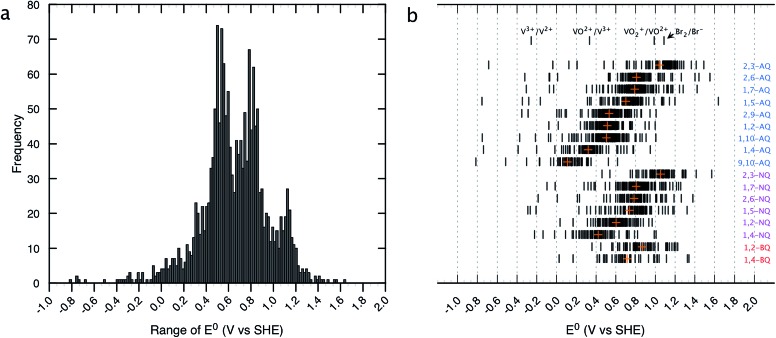
Distribution of HT screened quinone/hydroquinone redox couples. (a) A histogram of theoretically predicted *E* ^0^ (V *vs.* SHE). Number of bins and width of the histogram are 130 and 0.1 V, respectively. (b) *E* ^0^ windows for the different classes of quinones. The orange crosses show the mean values of *E* ^0^ for each class. Redox potentials of the conventional inorganic AFB redox couples are noted for comparison.^43^

The distribution of the predicted *E* ^0^ values between the Q/QH_2_ redox couples for the entire list of compounds existing in the virtual chemical library is shown in Fig. 2. By choosing Q/QH_2_ couples below 0.2 V for the negative and above 0.9 V for the positive sides of AFB, a practical battery voltage greater than 0.7 V is obtained. Based on the average *E* ^0^ values, the 9,10-AQ derived quinones are found to be most suitable for the negative side of AFBs, whereas the 1,2-BQ, 2,3-NQ, and 2,3-AQ derivatives are found to be appropriate for the positive side of AFBs.

**Fig. 3:**
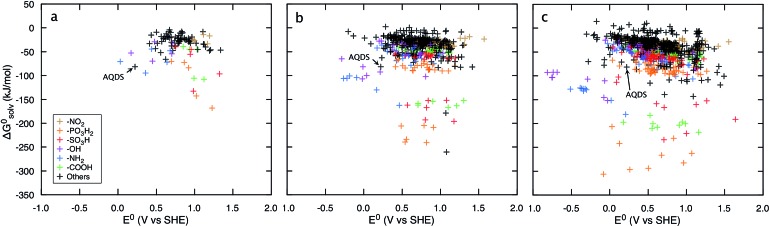
Redox-solubility maps of quinones of different sizes. *E* ^0^ and Δ*G*0solv distributions of (a) BQs, (b) NQs, and (c) AQs. The functional groups that effectively tune the *E* ^0^ and Δ*G*0solv are highlighted in color. AQDS molecule, which was successfully used for the negative side of an aqueous flow battery due to its low value of *E* ^0^ (0.222 V: calculated and 0.213 V: measured) as well as a high aqueous solubility (greater than 1 M at pH 0),^1^ is marked as reference point.

Fig. 3 shows that irrespective of the number of rings, functionalization of the quinones with the electron-donating groups (EDGs), such as –OH and –NH_2_, decreases the electron affinity of the compounds and results in low *E* ^0^ values. Functionalization of quinones with electron-withdrawing groups (EWGs), such as –SO_3_H, –PO_3_H_2_, and –NO_2_, shows an opposite effect and results in high *E* ^0^ values.^21^ Fig. 3 also shows that substituting quinone core hydrogens with any R-group lowers the Δ*G*0solv of the majority of the molecules. Thus, as a first approximation, we expect that the functionalized molecules would be more soluble than their parent quinone compounds. As shown in Fig. 3, for quinones of all sizes, incorporating the –OH, –NH_2_, –COOH, –SO_3_H, and –PO_3_H_2_ groups into the pure quinone backbones significantly decrease the Δ*G*0solv, and therefore these groups are predicted to increase the aqueous solubility the most. In particular, the hydrogen bonding ability, acidity, and polarity of the –SO_3_H and –PO_3_H_2_ groups are especially well-suited to achieve high aqueous solubility.^1,41,42^

### Structure–property relationships of quinones/hydroquinones

Structure–property relationships (SPRs) are useful for identifying the relationships between the new quinones and their chemical properties.^8^ SPRs provide beneficial information for fine-tuning the key properties of the candidate materials beyond the scope of the virtual library. In the present study, SPRs are identified quantitatively (*i.e.*, QSPRs) and they suggest: (*i*) general relationships between the core structures of different classes of quinones, the type of substituents, the positions of substituents, and their predicted properties; and (*ii*) the key substituents and their positions with respect to redox behaviors in a given quinone library.

**Fig. 4:**
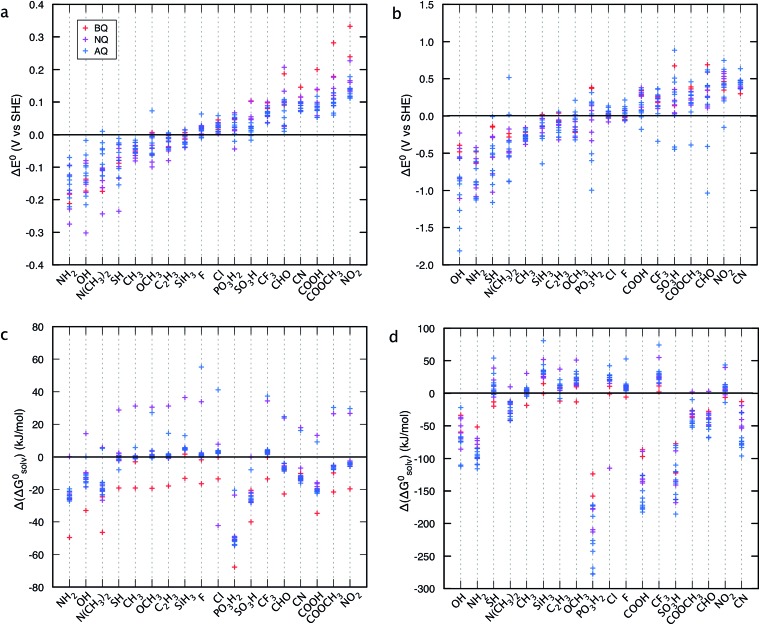
Effects of individual R-groups on tuning (a and b) the redox potential (*E* ^0^), and (c and d) the solubility (Δ*G* ^0^) of the parent quinone molecules. Panels a and c show the results when a quinone core hydrogen is substituted with an R-group, and panels b and d show the results when all the quinone core hydrogens are substituted with the same R-group.

Fig. 4 shows the change in theoretically predicted redox potentials (Δ*E* ^0^) and solvation free energies (Δ(Δ*G*0solv)) with respect to the pure parent quinones, after the quinones are functionalized with the R-groups. A positive value of Δ*E* ^0^ shows an increase in the redox potential, whereas a negative value of Δ(Δ*G*0solv) shows an increase in the solubility. The calculation methodology is given in ESI, Fig. S2.† Fig. 4a and b show the Δ*E* ^0^ between the possible isomers of BQs, NQs, and AQs and their 18 different R-group functionalized forms. Fig. 4a shows the Δ*E* ^0^ when the quinone/hydroquinone couples are decorated with a single R-group. For the parent quinones with multiple core hydrogen atoms that can be substituted in turn with a unique R-group, the Δ*E* ^0^ shows the mean value of the change in *E* ^0^ for all the possible substitutions.

Similarly, Fig. 4b shows the Δ*E* ^0^ when the quinone/hydroquinone couples are functionalized fully *via* the substitution of all the quinone core hydrogens for the same R-group. The R-groups in the abscissa of Fig. 4 are ordered according to increasing mean value of Δ*E* ^0^ of the same R-group substituted Q/QH_2_ molecules.

When compared to single substitutions, wider windows of *E* ^0^ and Δ*G*0solv are achieved by substituting all of the quinone core hydrogens with R-groups (Fig. 4b and d). As shown in Fig. 4c and d the side chains of –OH, –NH_2_, –COOH, –SO_3_H, and –PO_3_H_2_ are useful in increasing the water solubility of the quinones. The ordering of functional groups correlates with the nature of EDGs and EWGs, and trends from molecular induction theories and Hammett parameters.^44^ EDGs, such as –OH and –NH_2_, decrease the *E* ^0^ significantly when compared to their parent quinones with no side chains. Conversely, EWGs, such as –SO_3_H, –PO_3_H_2_, and –NO_2_ are useful in increasing the *E* ^0^.

**Fig. 5:**
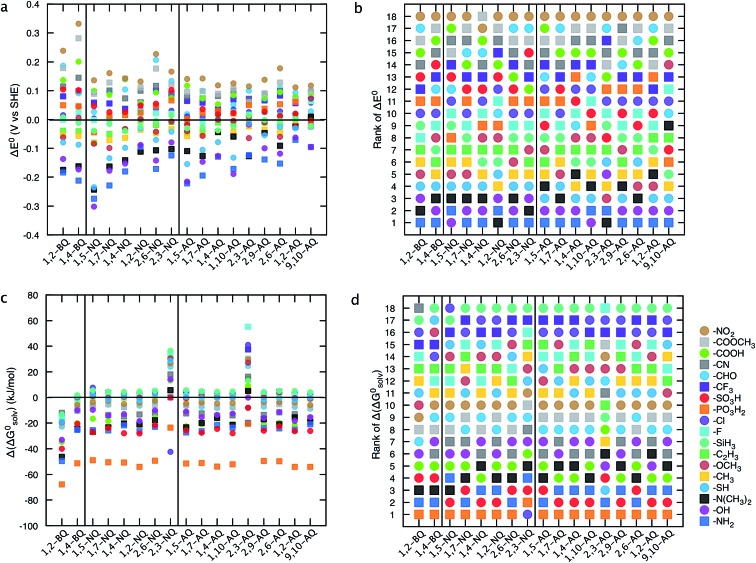
Effects of single R-group substitutions on tuning the redox potential and the solubility of different quinone classes. (a) Change in redox potential, Δ*E* ^0^, (c) change in solvation free energy, Δ(Δ*G*0solv) for only a single R-group functionalized quinones. The mean values are reported when the functional group is substituted in turn with different core hydrogens of a parent quinone. (b and d) Ranking of substituents in affecting the *E* ^0^ and Δ*G*0solv. The ranking is based on the efficiency of R-groups on decreasing the *E* ^0^ (b) or Δ*G*0solv (d). The results of the fully functionalized quinones are shown in ESI, Fig. S3.†

Fig. 5 provides a thorough investigation of the gross features of single R-group functionalized quinones. As shown in Fig. 5a and b irrespective of the class of quinone, EDGs groups, such as –OH, –NH_2_, and –N(CH_3_)_2_, are effective in decreasing the *E* ^0^, whereas EWGs, such as –COOH, –CHO, –PO_3_H_2_, –COOCH_3_, –SO_3_H, –CF_3_, –CN, and –NO_2_ are useful in increasing the *E* ^0^. The effects of EDGs on decreasing the *E* ^0^ are particularly notable for the class of 1,5-NQ, whereas the effects of EWGs on increasing the *E* ^0^ are remarkable especially for both of the two classes of BQs.

Functionalization of quinones with –PO_3_H_2_, –SO_3_H, –NH_2_, and –N(CH_3_)_2_ groups is expected to increase the predicted solubility of all quinone classes (Fig. 5c). Due to the simple solvation model employed and the fact that solid-state structures are not considered, the solubility predictions are qualitative, yet serve as a guiding tool for experimental synthesis. Fig. 5b and d show the ranking of the R-groups in decreasing the redox potential and increasing the solubility of the parent quinones. The HT analysis of the effects of R-group substitutions on the *E* ^0^ and Δ*G*0solv shown in Fig. 5 effectively captures the features of R-group functionalization for each different class of quinones and provides a valuable primary guide for the future synthesis of the new compounds.

**Fig. 6:**
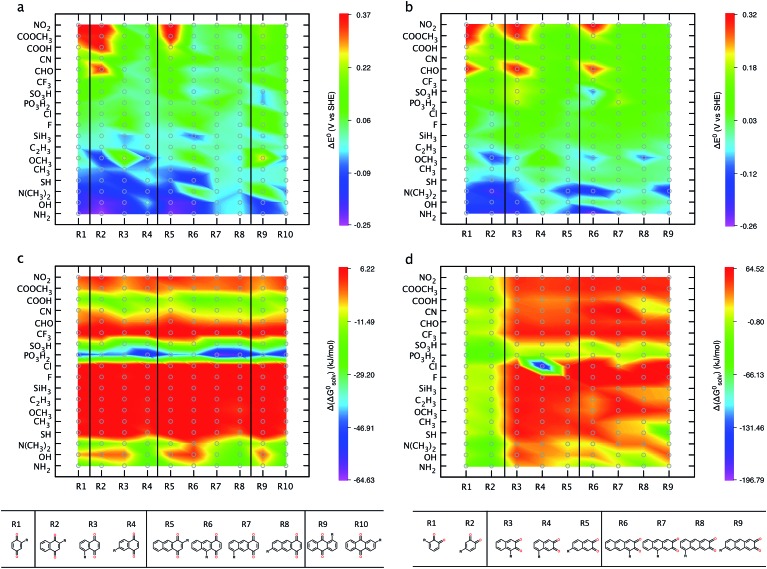
Positional effects of R-groups on the *E* ^0^ and Δ*G*0solv. (a and c) Show the results for the single R-group functionalized *para*-(BQ, NQs, and AQs), whereas (b and d) show the results for the single R-group functionalized *ortho*-(BQs, NQs, and AQs).

A critical factor in tuning the electrochemical properties of quinones is the position of the substituted groups on the quinone backbones.^4,22,23^ As a paradigmatic example, Fig. 6 shows the position effects on the *E* ^0^ and the (Δ*G*0solv) of the R-group functionalized *para*- and *ortho*-quinones of multiple rings. Fig. 6a shows that –NH_2_ and –OH functional groups are efficient in decreasing the redox potential. Our further investigation of the position of the newly incorporated side groups shows that the *E* ^0^ is remarkably affected by the distance between the newly substituted units and the cyclic ketone units of parent quinones.

As shown in Fig. 6a, incorporation of –NH_2_ or –OH into the *para*-quinones lowers the *E* ^0^ with the following order: R2 ≫ R3 > R4 in 1,4-NQ, R5 ≫ R6 > R7 ∼ R8 in 1,4-AQ, and R9 > R10 in 9,10-AQ. A similar positional dependency of *E* ^0^ is evident for the –NH_2_ and –OH substituted *ortho*-quinones (Fig. 6b). In agreement with a previous experimental study,^45^ the substitution of –OH groups adjacent to the ketone decreases *E* ^0^ more effectively than their substitution with remote benzenoid ring hydrogens. Similarly, the effects of –CHO, –CN, –COOH, –COOCH_3_, and –NO_2_ groups on increasing the redox potential are increased when these groups are substituted for the quinone hydrogens adjacent to the ketone units, such as R2 in 1,4-NQ, R5 in 1,4-AQ, and R9 in 9,10-AQ (Fig. 6a), and similarly for *ortho*-quinones R1 in 1,2-BQ, R3 in 2,3-NQ, and R6 in 2,3-AQ (Fig. 6b).

Fig. 6c shows that for *para*-quinones, the substitutions of –NH_2_, –OH, –N(CH_3_)_2_, –PO_3_H_2_, –SO_3_H, and –COOH groups are predicted to improve the solubility, especially when they are placed further away from the cyclic ketone units of the parent quinones. Functionalization of the positions R4 in 1,4-NQ, R7 and R8 in 1,4-AQ, and R10 in 9,10-AQ, are predicted to have the greatest effect in decreasing the solvation free energies (*i.e.*, augmented solubility). As shown in Fig. 5c, substitutions of most R-groups in 2,3-NQ and 2,3-AQ increase the value of Δ*G*0solv, thus the positional effects on the Δ*G*0solv are not clearly visible (Fig. 6d).

In an effort to guide future work on small organic molecule based AFBs, here, we summarize the key findings of our HT screening and QSPR analysis. First, we identified 104 Q/QH_2_ couples with *E* ^0^ lower than 0.2 V *vs.* SHE, and 304 Q/QH_2_ couples with *E* ^0^ higher than 0.9 V *vs.* SHE. As a reference data, the full list of interesting candidate molecules is given in ESI, Table S1.† Using the key findings of our HT screening, QSPR analysis, and our own chemical intuition of some of the expected behaviors of substituted quinones, such as the stability in water solution and the synthetic feasibility, we identified a shorter list of compounds. We also focused on compounds that have solvation free energy below a certain threshold (*i.e.* <–81.5 kJ mol^–1^, that is the calculated value of AQDS's solvation free energy in water.^1^) As a result, we shortlisted 31 quinones with redox potentials < 0.2 V and 28 quinones with > 1.0 V. These highly promising molecules are marked with an asterisk in ESI, Table S1.† Second, the class of 9,10-AQs is a suitable target for the negative side of an AFB cell, whereas the classes of 1,2-BQs, 2,3-NQs, and 2,3-AQs are more appropriate for the positive side of the cell (Fig. 1b). Third, utilizing EDGs (–OH and –NH_2_) decreases *E* ^0^ of the pure quinones, thus making them more attractive for the negative side of an AFB. In contrary, utilizing EWGs (–SO_3_H, –PO_3_H_2_, and –NO_2_) increases *E* ^0^ of the parent quinones, thus making them more attractive for the positive side of an AFB (Fig. 4a and b). Fourth, the effects of EDGs, such as –NH_2_ and –OH, in decreasing the *E* ^0^ are increased when these groups are positioned close to the quinone ketone groups. Similarly, the effects of EWGs, such as –NO_2_, –COOCH_3_, –COOH, –CN, and –CHO, in increasing the *E* ^0^ are increased when they are positioned close to the ketone groups (Fig. 6a and b). Fifth, substitutions of hydrophilic groups such as –OH, –NH_2_, –COOH, –SO_3_H, and –PO_3_H_2_ are predicted to increase the solubility. In general, full substitutions are more useful than single substitutions in improving the water solubility (Fig. 4c and d). The highly substituted quinones may have relatively lower synthetic accessibility due to steric hindrance. Last, good aqueous solubility can be achieved by substituting quinone hydrogens that are far from the quinone ketone groups with hydrophilic functional groups, such as –SO_3_H, and –PO_3_H_2_ (Fig. 6c and d).

### Stability of molecules for energy storage

Given the huge number of organic molecules to be screened, we utilized HT computational screening strategies to reduce the search space of substituted quinones in this study. The ultimate goal of this study is to discover promising molecules for long-term energy storage. Stability is important not only to prevent chemical loss for long cycle life, but also because reaction with the electrode can compromise the electrode's conductivity and surface area.

From a system's point of view, the use of quinones offers an advantage in stability over current flow battery technologies, because the quinone can exhibit minimal membrane crossover.^1^ On the other hand, the use of oxidizing quinones generates the possibility for decomposition reactions such as polymerization and oxidation of the carbon backbone. These limitations can be mitigated by replacing C–H groups adjacent to C=O groups (vulnerable to oxidation) with groups such as C–R, where R is hydroxyl, sulfonyl, amino, or carboxyl.

From the computation point of view, predicting the stability of a molecule is challenging because of the many possible decomposition reactions and products. These sort of computations can be implemented in HT screening by identifying and screening for indicators of reactivity, which would initially be identified through experiments. One way that the stability of a molecule can be quantified is by calculating the rate at which it goes over the lowest barrier separating it from its isomerization or dissociation products. Alternatively, comparison of the frontier orbitals (*i.e.* the highest occupied molecular orbital, HOMO, and the lowest unoccupied molecular orbital, LUMO) or Fukui functions^46^ of the new molecules with those of stable quinones (such as AQDS) could predict their reactivity with electrophiles and nucleophiles, because—to a first approximation—the reactivity will be governed by the energies of these orbitals. Decomposition pathways due to solvent pH effects and electrical bias are also important future research directions.

## Conclusions

We demonstrate the use of robust HT theoretical calculations as a fast screening tool for the discovery and the future design of potentially interesting quinone molecules for organic-based AFBs. The QSPR analysis presented in this study underlies the use of AQDS/AQDSH_2_ organic couple at the negative side of an AFB in our recent study.^1^ In this study, the predicted *E* ^0^ values of Q/QH_2_ redox couples serve as a pivotal reference data for the target electrochemical window of a full-cell organic AFB. Moreover, the key finding that functionalization near to the ketones impacts reduction potential and away from the ketone improves solubility will be critical to further tuning the properties of new quinone electrolytes. The identification of over 300 quinones with a predicted reduction potential above 0.7 V and the refined list of synthetically feasible quinones should lead the way to a high-performance all-quinone flow battery, which is predicted to vastly reduce the cost of electrical energy storage.

## Author contributions

SE and CS contributed equally to this work. AAG conceived the project. SE and CS carried out the research, with direction from AAG. SE and CS were responsible for the generation of candidate materials library, theoretical screening of the candidate materials, and managing of results. SE and CS carried out the structural analysis of the top candidates with contributions from MPM. MPM's input was crucial for experimental assessment and for keeping a close loop with experimental parameters. SE and CS wrote the manuscript with contributions from MPM and AAG.
