# Supplementary material for: Computational design of molecules for an all-quinone redox flow battery
Source: Chem Sci. 2014 Nov 21;6(2):885–93. doi: 10.1039/c4sc03030c (PMC5811157; doi:10.1039/c4sc03030c)
Supplement: Supplementary file 1 [file SC-006-C4SC03030C-s001.pdf]

Electronic Supplementary Material (ESI) for Chemical Science.
This journal is © The Royal Society of Chemistry 2014

† Electronic Supplementary Information (ESI)

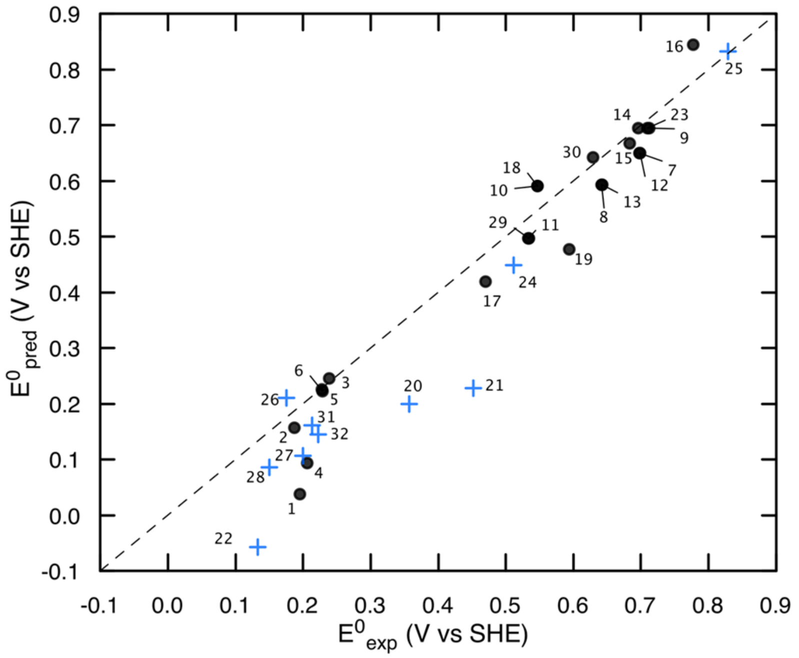

Fig. S1 Computationally predicted vs. experimentally measured E^0. The experimental E^0 values are compiled from ref. 1-5 in ESI. Black circles represent aqueous measurements and blue crosses represent measurements in alcohol solution. The data points represent the following quinone compounds: 1. AQ-1-sulfonic acid, 2. AQ-2-sulfonic acid, 3. AQ-1,5-disulfonic acid, 4. AQ-1,8-disulfonic acid, 5. AQ-2,6-disulfonic acid, 6. AQ-2,7-disulfonic acid, 7. Benzoquinone, 8. Toluquinone, 9. Chloroquinone, 10. 1,2-naphthoquinone, 11. 1,4-naphthoquinone-3-sulfonic acid, 12. Benzoquinone, 13. Toluquinone, 14. Chloroquinone, 15. Chloranil (tetrachloroquinone), 16. o-benzoquinone, 17. 1,4-naphthoquinone, 18. 1,2-naphthoquinone, 19. Hydroxy-benzoquinone, 20. 2-hydroxy-1,4-naphthoquinone, 21. 8-hydroxy-1,4-naphthoquinone, 22. 1-hydroxy-anthraquinone, 23. Chlorobenzoquinone, 24. 2-chloro-1,4-naphthoquinone, 25. Tetrachloro-o-benzoquinone, 26. 1-chloro-anthraquinone, 27. 2-chloro-anthraquinone, 28. 2-methyl-anthraquinone, 29. 1,4-naphthoquinone-2-sulfonic acid, 30. 1,2-naphthoquinone-4-sulfonic acid, 31. Anthraquinone-2-carboxylic acid, 32. Methyl anthraquinone-2-carboxylate.

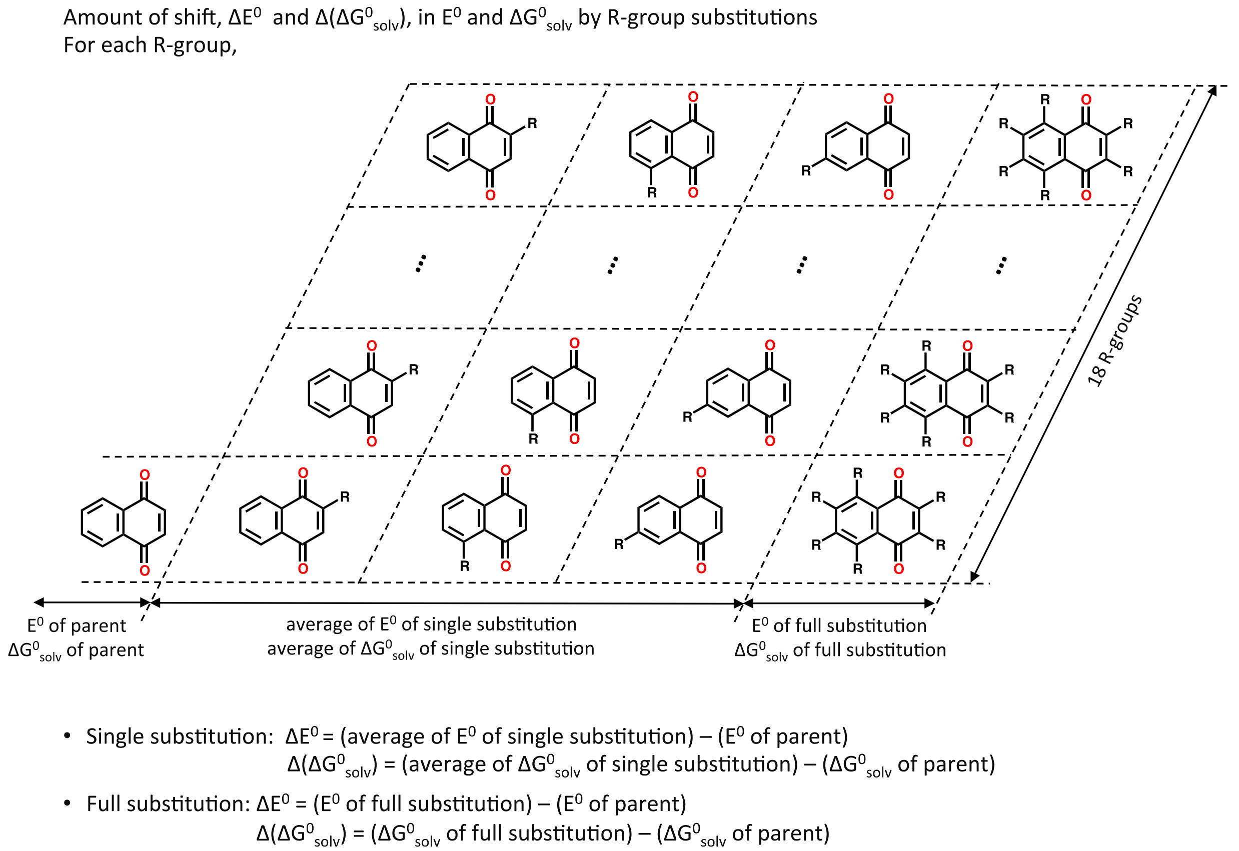

Fig. S2 Change in E^0 and ΔG^0_{solv} as a result of functionalization with R-groups. To understand the effects of functional groups on tuning the redox properties and the solubility of the parent molecules here we introduce two new terms, ΔE^0 and $\Delta(\Delta G^0_{solv})$. The ΔE^0 and $\Delta(\Delta G^0_{solv})$ show the difference between the E^0 and ΔG^0_{solv} of R-group decorated Q/QH$_2$ couples and the E^0 and ΔG^0_{solv} of pure parent Q/QH$_2$ couples, respectively. As an example, 1,4-NQ molecule is shown. Note that for single R-group substituted quinones the mean values of change in E^0 and ΔG^0_{solv} are reported.

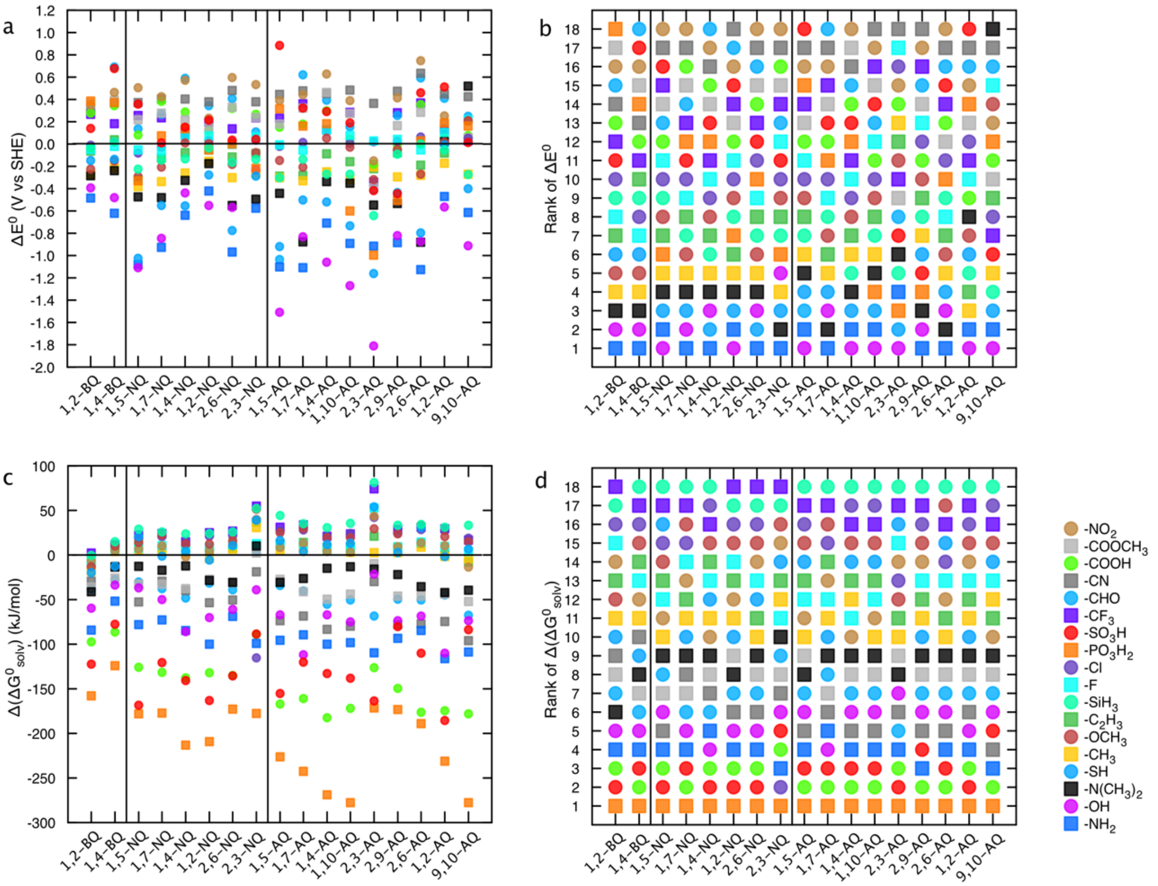

Fig. S3 Effects of full R-group substitutions on tuning the redox and the solubility of different quinone classes. (a) Change in redox potential, ΔE^0, and **c**, change in solvation free energy, $\Delta(\Delta G^0_{solv})$ for fully functionalized quinone molecules. (b and d) Ranking of substituents in affecting the E^0 and ΔG^0_{solv}. The ranking is based on the efficiency of R-groups on decreasing the E^0 (b) or ΔG^0_{solv} (d).

Table S1 The list of computationally predicted candidate quinone molecules with interesting redox properties, i.e., $E^0 < 0.2$ V vs. SHE and $E^0 > 0.9$ V vs. SHE. Note that the refined list is based on the key findings of our HT screening, QSPR analysis, expected behaviors of substituted quinone compounds (see main text for details).

ID	Class	R-group substituted	Position of substituted R-group 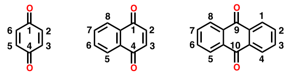 Numbering convention	ΔG^0_{solv} (kJ/mol)	E^0 (V vs SHE)	Short list
1	9,10-AQ	OH	Full	-92.83	-0.81	*
2	1,5-AQ	OH	Full	-103.41	-0.75	*
3	1,10-AQ	OH	Full	-103.53	-0.75	*
4	1,4-AQ	OH	Full	-92.67	-0.74	*
5	2,3-AQ	OH	Full	-92.66	-0.69	*
6	9,10-AQ	NH_2	Full	-127.84	-0.51	*

7	1,4-AQ	NH_2	Full	-125.38	-0.39	*
8	1,10-AQ	NH_2	Full	-126.65	-0.37	*
9	2,9-AQ	NH_2	Full	-127.01	-0.35	*
10	1,5-AQ	NH_2	Full	-131.98	-0.34	*
11	2,6-AQ	NH_2	Full	-126.90	-0.32	*
12	1,7-AQ	NH_2	Full	-129.44	-0.30	*
13	9,10-AQ	SH	Full	-12.62	-0.30	
14	1,5-NQ	OH	Full	-64.95	-0.29	
15	2,9-AQ	OH	Full	-107.10	-0.29	*
16	1,5-AQ	CHO	Full	-70.39	-0.28	
17	1,5-NQ	NH_2	Full	-106.22	-0.26	*
18	1,4-NQ	NH_2	Full	-105.60	-0.22	*
19	1,10-AQ	SH	Full	-15.64	-0.21	
20	1,5-NQ	SH	Full	-7.56	-0.20	
21	1,4-AQ	SH	Full	-13.57	-0.20	
22	2,6-NQ	NH_2	Full	-100.88	-0.18	*
23	9,10-AQ	SiH_3	Full	14.03	-0.17	
24	9,10-AQ	CH_3	Full	-23.52	-0.17	
25	1,5-AQ	SH	Full	-19.99	-0.16	
26	1,4-NQ	SH	Full	-17.02	-0.14	
27	1,7-NQ	NH_2	Full	-104.51	-0.09	*
28	1,10-AQ	PO_3H_2	Full	-306.07	-0.08	*
29	1,2-AQ	OH	Full	-145.40	-0.08	*
30	2,6-AQ	$N(CH_3)_2$	Full	-77.85	-0.07	
31	1,7-AQ	$N(CH_3)_2$	Full	-66.29	-0.07	
32	2,6-AQ	OH	Full	-110.54	-0.07	*
33	9,10-AQ	OH	R1	-24.99	-0.06	
34	1,10-AQ	OH	R9	-24.97	-0.06	
35	2,3-AQ	SH	Full	-16.95	-0.04	
36	1,7-AQ	OH	Full	-151.92	-0.03	*
37	9,10-AQ	NH_2	R1	-32.43	-0.03	
38	1,4-NQ	OH	Full	-106.88	-0.02	*
39	1,4-AQ	$N(CH_3)_2$	Full	-40.63	-0.01	
40	1,7-NQ	OH	Full	-81.74	-0.01	*
41	2,9-AQ	$N(CH_3)_2$	Full	-55.34	0.00	
42	2,6-NQ	SH	Full	-21.18	0.01	
43	2,6-AQ	SH	Full	-11.45	0.01	
44	9,10-AQ	$N(CH_3)_2$	R2	-41.86	0.01	
45	1,2-AQ	NH_2	Full	-151.34	0.02	*
46	9,10-AQ	OCH_3	R2	-21.19	0.02	
47	1,4-BQ	NH_2	Full	-70.68	0.03	

48	2,9-AQ	PO_3H_2	Full	-206.50	0.03	*
49	9,10-AQ	PO_3H_2	R1	-67.02	0.03	
50	9,10-AQ	NH_2	R2	-47.79	0.04	
51	9,10-AQ	SO_3H	R1	-39.50	0.04	
52	1,2-NQ	OH	Full	-100.13	0.04	*
53	9,10-AQ	SiH_3	R1	-14.30	0.04	
54	2,9-AQ	NH_2	R1	-33.42	0.05	
55	1,4-AQ	SiH_3	Full	5.07	0.05	
56	1,10-AQ	SH	R9	-18.42	0.06	
57	9,10-AQ	SH	R1	-13.91	0.07	
58	9,10-AQ	CH_3	R1	-17.46	0.07	
59	9,10-AQ	OH	R2	-43.85	0.07	
60	1,4-AQ	CH_3	Full	-19.38	0.07	
61	9,10-AQ	SH	R2	-24.23	0.08	
62	9,10-AQ	CH_3	R2	-20.89	0.09	
63	9,10-AQ	C_2H_3	R2	-20.86	0.09	
64	2,9-AQ	SO_3H	Full	-113.61	0.09	*
65	1,4-AQ	NH_2	R2	-46.84	0.09	
66	1,4-NQ	$N(CH_3)_2$	Full	-33.30	0.09	
67	9,10-AQ	F	R2	-17.61	0.10	
68	9,10-AQ	None	R0	-19.10	0.10	
69	2,9-AQ	SH	Full	-31.06	0.10	
70	9,10-AQ	CHO	R1	-28.35	0.10	
71	9,10-AQ	Cl	R2	-15.62	0.11	
72	1,4-AQ	OH	R2	-31.65	0.11	
73	9,10-AQ	SO_3H	Full	-102.71	0.11	*
74	9,10-AQ	SiH_3	R2	-15.68	0.12	
75	9,10-AQ	C_2H_3	R1	-18.54	0.13	
76	9,10-AQ	PO_3H_2	R2	-79.61	0.13	
77	1,10-AQ	OH	R4	-28.75	0.13	
78	2,3-AQ	PO_3H_2	Full	-242.19	0.13	*
79	1,4-AQ	OH	R10	-28.63	0.13	
80	9,10-AQ	CF_3	Full	-2.62	0.13	
81	1,4-AQ	NH_2	R10	-39.42	0.13	
82	1,4-AQ	SH	R10	-22.56	0.14	
83	9,10-AQ	$COOCH_3$	R2	-24.22	0.15	
84	9,10-AQ	CF_3	R2	-16.27	0.15	
85	9,10-AQ	Cl	Full	-0.40	0.15	
86	9,10-AQ	C_2H_3	Full	-27.04	0.15	
87	1,10-AQ	NH_2	R9	-42.91	0.15	
88	9,10-AQ	CHO	R2	-27.38	0.15	

89	9,10-AQ	$COOCH_3$	Full	-71.13	0.15	
90	9,10-AQ	SO_3H	R2	-51.02	0.16	
91	9,10-AQ	CN	R2	-32.05	0.16	
92	1,4-NQ	CH_3	Full	-19.33	0.16	
93	9,10-AQ	COOH	R2	-41.39	0.16	
94	1,2-NQ	NH_2	Full	-130.08	0.17	*
95	1,4-BQ	OH	Full	-52.90	0.17	
96	1,10-AQ	$N(CH_3)_2$	Full	-41.67	0.17	
97	1,4-NQ	NH_2	R2	-42.29	0.17	
98	9,10-AQ	COOH	Full	-197.31	0.18	*
99	1,4-AQ	$N(CH_3)_2$	R2	-44.59	0.18	
100	9,10-AQ	NO_2	R2	-22.64	0.18	
101	1,10-AQ	SH	R4	-21.90	0.19	
102	9,10-AQ	CF_3	R1	-18.78	0.19	
103	1,4-NQ	OH	R2	-27.79	0.20	
104	1,10-AQ	CH_3	Full	-25.74	0.20	
105	1,2-BQ	Cl	R6	-24.76	0.90	
106	1,5-NQ	CF_3	R4	-25.75	0.90	
107	1,2-AQ	CHO	Full	-80.10	0.90	
108	1,7-NQ	CN	R5	-46.70	0.90	
109	1,7-NQ	COOH	R6	-50.57	0.90	
110	1,7-AQ	PO_3H_2	R10	-90.30	0.90	
111	2,3-NQ	SiH_3	Full	-11.46	0.90	
112	1,7-AQ	CN	R3	-50.19	0.90	
113	1,5-NQ	COOH	Full	-154.07	0.90	
114	2,6-AQ	CF_3	R10	-35.27	0.90	
115	1,5-NQ	NO_2	R3	-29.44	0.90	
116	2,3-AQ	C_2H_3	Full	-49.99	0.90	
117	1,7-NQ	COOH	R4	-46.52	0.91	
118	1,7-NQ	PO_3H_2	Full	-208.86	0.91	
119	1,5-AQ	COOH	Full	-203.41	0.91	
120	1,10-AQ	NO_2	Full	-25.43	0.91	
121	1,7-NQ	CF_3	R2	-29.99	0.91	
122	1,5-NQ	CN	R2	-25.29	0.91	
123	2,6-AQ	NO_2	R10	-42.26	0.91	
124	2,3-NQ	NH_2	R6	-74.89	0.91	
125	1,5-NQ	COOH	R4	-39.04	0.91	
126	1,7-AQ	SO_3H	R6	-79.17	0.92	
127	1,7-NQ	SO_3H	R2	-49.66	0.92	
128	2,6-NQ	NO_2	R4	-32.96	0.92	
129	2,3-AQ	CH_3	Full	-68.22	0.92	

130	1,2-BQ	$COOCH_3$	R5	-29.30	0.92
131	2,6-NQ	CF_3	Full	-5.38	0.92
132	1,2-BQ	CF_3	R5	-21.62	0.92
133	1,2-BQ	PO_3H_2	R5	-83.76	0.92
134	2,6-NQ	CN	R5	-42.07	0.92
135	1,7-NQ	NO_2	R4	-29.02	0.92
136	1,7-NQ	CN	R4	-37.93	0.93
137	2,6-AQ	CN	R5	-53.80	0.93
138	1,7-NQ	CF_3	R4	-23.67	0.93
139	1,7-AQ	CN	R8	-57.23	0.93
140	1,7-NQ	PO_3H_2	R4	-88.13	0.93
141	1,2-BQ	CF_3	R6	-26.25	0.93
142	1,7-NQ	CF_3	R8	-32.93	0.93
143	2,3-AQ	$N(CH_3)_2$	R4	-77.08	0.93
144	2,6-AQ	CN	R10	-48.76	0.93
145	1,4-BQ	$COOCH_3$	R2	-28.89	0.93
146	1,2-BQ	COOH	R5	-44.99	0.93
147	1,7-NQ	CN	R3	-40.04	0.93
148	1,2-NQ	CHO	Full	-61.77	0.94
149	1,5-AQ	$COOCH_3$	Full	-63.41	0.94
150	1,2-AQ	CN	Full	-109.85	0.94
151	2,3-AQ	$N(CH_3)_2$	R6	-75.52	0.94
152	2,6-AQ	CHO	R10	-48.50	0.94
153	1,5-NQ	CN	R4	-41.77	0.94
154	1,2-BQ	SO_3H	R5	-55.33	0.94
155	2,6-AQ	SO_3H	R10	-67.28	0.94
156	2,3-NQ	$N(CH_3)_2$	R4	-51.82	0.95
157	1,7-AQ	CF_3	R8	-38.76	0.95
158	1,2-BQ	CHO	R5	-32.21	0.95
159	2,3-AQ	COOH	Full	-197.14	0.95
160	2,6-NQ	CF_3	R5	-26.78	0.95
161	2,9-AQ	NO_2	Full	-24.14	0.95
162	1,4-AQ	NO_2	Full	-21.45	0.95
163	2,3-NQ	C_2H_3	Full	-26.49	0.95
164	2,6-AQ	CF_3	R5	-36.97	0.95
165	1,2-BQ	CN	R5	-25.49	0.95
166	1,2-BQ	SO_3H	R6	-45.23	0.95
167	1,5-NQ	CHO	Full	-58.34	0.95
168	1,7-NQ	CN	R2	-47.40	0.96
169	2,6-AQ	PO_3H_2	R10	-92.93	0.96
170	2,3-NQ	SH	R4	-24.59	0.96

171	1,7-AQ	SO_3H	R8	-76.95	0.96	
172	2,3-NQ	OCH_3	Full	-12.30	0.96	
173	1,5-NQ	COOH	R2	-45.53	0.96	
174	1,7-NQ	NO_2	R8	-37.24	0.96	
175	1,7-AQ	PO_3H_2	Full	-282.49	0.97	
176	1,2-BQ	CN	R6	-19.04	0.97	
177	1,7-NQ	NO_2	R3	-30.30	0.97	
178	1,2-NQ	CN	Full	-83.43	0.97	
179	2,3-NQ	OCH_3	R4	-25.02	0.97	
180	2,3-NQ	OH	R6	-59.23	0.97	
181	2,3-AQ	NO_2	Full	-27.48	0.97	
182	1,7-AQ	CHO	R6	-47.91	0.97	
183	2,3-NQ	$N(CH_3)_2$	R5	-50.00	0.97	
184	1,7-AQ	CN	R9	-55.17	0.98	
185	1,4-BQ	NO_2	R2	-24.91	0.98	
186	1,2-BQ	SO_3H	Full	-132.64	0.98	
187	1,7-AQ	COOH	Full	-200.68	0.99	
188	2,3-NQ	SH	R6	-42.58	0.99	
189	1,5-AQ	CF_3	Full	-5.36	0.99	
190	2,3-NQ	NH_2	R5	-56.82	0.99	
191	1,7-NQ	$COOCH_3$	R8	-40.30	0.99	
192	2,3-NQ	OCH_3	R6	-41.65	0.99	
193	1,4-NQ	NO_2	Full	-23.34	0.99	
194	1,7-NQ	COOH	R8	-57.70	0.99	
195	1,4-BQ	COOH	Full	-105.46	0.99	
196	1,7-NQ	CN	R8	-51.42	0.99	
197	2,3-NQ	CH_3	R4	-29.76	0.99	
198	2,3-NQ	C_2H_3	R6	-35.45	1.00	
199	1,2-BQ	NO_2	R5	-26.41	1.00	
200	2,3-NQ	CH_3	R6	-34.40	1.00	
201	1,2-AQ	SO_3H	Full	-220.91	1.00	
202	1,7-NQ	COOH	R2	-47.88	1.00	
203	2,6-NQ	CHO	R3	-38.26	1.00	
204	1,10-AQ	CN	Full	-108.22	1.00	
205	2,3-AQ	OCH_3	R5	-36.76	1.01	
206	1,7-AQ	$COOCH_3$	R6	-48.88	1.01	
207	1,4-NQ	CHO	Full	-69.27	1.01	
208	2,9-AQ	CN	Full	-111.60	1.01	
209	2,6-NQ	$COOCH_3$	R5	-37.30	1.01	
210	1,7-AQ	NO_2	R6	-51.73	1.01	
211	2,3-AQ	SO_3H	R4	-97.32	1.01	*

212	2,6-NQ	$COOCH_3$	R3	-39.30	1.02	
213	2,3-NQ	CH_3	R5	-32.60	1.02	
214	1,4-BQ	CN	Full	-32.01	1.02	
215	2,3-AQ	NH_2	R10	-76.97	1.02	
216	1,4-BQ	PO_3H_2	Full	-142.99	1.02	*
217	2,3-NQ	PO_3H_2	R6	-90.20	1.02	*
218	2,3-AQ	OCH_3	R4	-60.41	1.02	
219	2,6-AQ	$COOCH_3$	R3	-50.61	1.03	
220	1,2-BQ	COOH	R6	-45.00	1.03	
221	2,6-AQ	$COOCH_3$	R5	-46.68	1.03	
222	2,3-NQ	OH	R5	-53.26	1.03	
223	1,5-NQ	$COOCH_3$	Full	-60.42	1.03	
224	2,3-NQ	C_2H_3	R5	-31.92	1.03	
225	2,3-AQ	NH_2	R6	-78.20	1.04	
226	1,7-AQ	$COOCH_3$	R8	-48.17	1.04	
227	2,3-NQ	None	R0	-63.41	1.04	
228	2,3-NQ	F	R6	-32.13	1.04	
229	2,6-AQ	COOH	R3	-66.75	1.04	
230	1,4-BQ	$COOCH_3$	Full	-45.18	1.04	
231	2,3-NQ	Cl	R6	-29.71	1.04	
232	2,3-NQ	SH	R5	-36.84	1.04	
233	2,3-AQ	$N(CH_3)_2$	R10	-54.74	1.04	
234	1,7-NQ	$COOCH_3$	R6	-39.52	1.04	
235	2,3-NQ	SiH_3	R6	-27.91	1.05	
236	2,3-NQ	SiH_3	R5	-27.24	1.05	
237	2,6-AQ	NO_2	R3	-52.53	1.05	
238	2,3-NQ	C_2H_3	R4	-29.14	1.05	
239	2,6-NQ	NO_2	R5	-31.89	1.06	
240	2,3-NQ	SiH_3	R4	-25.48	1.06	
241	2,3-NQ	F	R4	-27.73	1.06	
242	2,6-NQ	NO_2	R3	-38.50	1.06	
243	1,7-NQ	NO_2	R6	-40.20	1.06	
244	1,5-AQ	COOH	R2	-59.88	1.06	
245	1,7-NQ	CF_3	Full	-7.66	1.07	
246	1,5-AQ	PO_3H_2	Full	-262.40	1.07	*
247	2,6-AQ	CHO	R5	-41.60	1.07	
248	2,3-NQ	Cl	Full	-178.62	1.07	
249	1,5-NQ	CF_3	Full	-5.83	1.07	
250	2,3-NQ	F	R5	-29.05	1.07	
251	2,3-NQ	$COOCH_3$	R6	-35.61	1.07	
252	2,6-NQ	CHO	R5	-32.63	1.07	

253	1,5-AQ	$COOCH_3$	R2	-47.85	1.07	
254	2,3-AQ	$N(CH_3)_2$	R5	-55.55	1.08	
255	2,3-AQ	C_2H_3	R6	-65.29	1.08	
256	2,3-NQ	Cl	R5	-260.20	1.08	
257	2,3-NQ	CHO	R6	-40.16	1.08	
258	2,3-NQ	SO_3H	Full	-151.95	1.08	*
259	1,7-NQ	CHO	R8	-43.65	1.08	
260	2,3-NQ	Cl	R4	-27.23	1.08	
261	2,3-AQ	CH_3	R4	-47.82	1.08	
262	1,5-AQ	NO_2	R2	-45.47	1.08	
263	2,3-NQ	COOH	R6	-51.58	1.08	
264	2,3-AQ	NH_2	R5	-106.45	1.08	*
265	1,7-AQ	NO_2	R8	-44.55	1.08	
266	2,3-NQ	SO_3H	R6	-63.63	1.08	
267	2,3-AQ	OH	R6	-50.57	1.09	
268	2,3-AQ	OCH_3	R6	-44.44	1.09	
269	2,6-AQ	NO_2	R5	-43.62	1.09	
270	2,3-AQ	SH	R6	-143.02	1.09	*
271	2,6-AQ	$COOCH_3$	Full	-88.41	1.09	*
272	2,3-NQ	CF_3	R6	-29.09	1.09	
273	2,3-AQ	PO_3H_2	R4	-82.21	1.09	*
274	2,3-AQ	OH	R10	-87.13	1.10	*
275	1,7-NQ	CHO	Full	-69.52	1.10	
276	1,5-NQ	$COOCH_3$	R2	-35.28	1.10	
277	2,6-NQ	COOH	Full	-166.86	1.10	*
278	2,3-AQ	CH_3	R6	-73.86	1.10	
279	2,3-NQ	PO_3H_2	R5	-90.31	1.10	*
280	1,7-NQ	CHO	R2	-36.80	1.10	
281	2,3-NQ	F	Full	-50.86	1.10	
282	2,3-AQ	CH_3	R10	-65.75	1.11	
283	1,7-NQ	$COOCH_3$	Full	-64.72	1.11	
284	1,2-BQ	CF_3	Full	-8.19	1.11	
285	2,3-NQ	$COOCH_3$	R5	-38.64	1.11	
286	2,3-AQ	CH_3	R5	-73.37	1.11	
287	2,3-NQ	CF_3	R5	-29.36	1.11	
288	1,4-BQ	NO_2	Full	-14.08	1.11	
289	1,2-BQ	CHO	R6	-33.91	1.11	
290	2,6-NQ	$COOCH_3$	Full	-62.91	1.11	
291	2,3-NQ	CN	R6	-45.01	1.11	
292	2,3-AQ	SH	R5	-69.72	1.12	
293	1,7-AQ	COOH	R2	-62.89	1.12	

294	2,3-AQ	OH	R4	-60.74	1.12	
295	1,2-BQ	COOH	Full	-107.66	1.12	*
296	2,3-NQ	COOH	R5	-52.71	1.12	
297	2,3-AQ	SH	R4	-38.21	1.12	
298	2,3-AQ	SH	R10	-64.60	1.12	
299	2,3-AQ	C_2H_3	R5	-62.45	1.12	
300	2,3-AQ	OH	R5	-85.42	1.12	*
301	2,3-AQ	OCH_3	R10	-33.54	1.12	
302	2,3-AQ	None	R0	-70.90	1.13	
303	1,2-BQ	$COOCH_3$	R6	-34.49	1.13	
304	2,3-NQ	COOH	R4	-46.41	1.13	
305	1,7-AQ	$COOCH_3$	R2	-52.04	1.13	
306	2,3-AQ	C_2H_3	R4	-62.45	1.13	
307	1,7-AQ	SO_3H	Full	-160.06	1.13	*
308	2,3-NQ	CHO	R5	-42.30	1.13	
309	2,3-AQ	C_2H_3	R10	-35.38	1.13	
310	2,3-AQ	NO_2	R6	-43.42	1.13	
311	2,3-NQ	OCH_3	R5	-32.03	1.13	
312	1,7-AQ	$COOCH_3$	Full	-81.20	1.13	
313	1,5-NQ	NO_2	R2	-33.06	1.13	
314	2,3-NQ	CN	R5	-46.02	1.13	
315	2,3-AQ	F	R6	-36.47	1.13	
316	2,3-AQ	SiH_3	R6	-66.25	1.13	
317	2,3-NQ	CN	R4	-45.20	1.13	
318	2,3-AQ	Cl	R6	-12.05	1.13	
319	2,3-AQ	SiH_3	R5	-63.10	1.13	
320	2,3-AQ	F	R4	-6.38	1.14	
321	2,3-NQ	NO_2	R6	-37.02	1.14	
322	2,3-NQ	SO_3H	R5	-63.48	1.14	
323	2,3-AQ	SiH_3	R10	-56.68	1.14	
324	1,5-AQ	NO_2	Full	-27.98	1.14	
325	1,2-BQ	CN	Full	-39.96	1.14	
326	2,3-NQ	CHO	Full	-60.62	1.14	
327	2,3-AQ	SO_3H	R6	-69.19	1.14	
328	2,3-AQ	CHO	R6	-46.24	1.15	
329	1,7-NQ	$COOCH_3$	R2	-42.94	1.15	
330	2,3-AQ	Cl	Full	-28.71	1.15	
331	2,3-AQ	SiH_3	R4	-45.47	1.15	
332	2,3-AQ	SO_3H	R5	-84.20	1.15	*
333	2,3-AQ	F	R5	-11.36	1.15	
334	2,3-AQ	$COOCH_3$	R5	-39.22	1.15	

335	2,3-AQ	Cl	R5	-32.21	1.15	
336	2,3-AQ	$COOCH_3$	R6	-41.25	1.15	
337	2,3-AQ	F	R10	-8.43	1.15	
338	2,3-AQ	$COOCH_3$	R10	-42.21	1.15	
339	2,3-AQ	PO_3H_2	R6	-95.90	1.15	*
340	2,3-AQ	F	Full	-18.27	1.15	
341	1,7-AQ	NO_2	R2	-48.56	1.16	
342	2,3-AQ	COOH	R5	-74.49	1.16	
343	2,3-AQ	COOH	R6	-56.78	1.16	
344	2,3-AQ	Cl	R10	-67.94	1.16	
345	2,3-AQ	Cl	R4	-6.90	1.16	
346	2,6-AQ	COOH	Full	-218.30	1.16	*
347	2,3-NQ	NO_2	R5	-38.96	1.16	
348	2,3-AQ	CF_3	R6	-34.09	1.17	
349	1,7-AQ	CF_3	Full	-6.40	1.17	
350	1,2-BQ	NO_2	R6	-33.43	1.17	
351	2,3-AQ	CHO	R5	-47.60	1.17	
352	2,3-NQ	CF_3	R4	-28.93	1.17	
353	2,3-AQ	CF_3	R5	-33.54	1.17	
354	2,6-AQ	CF_3	Full	-11.26	1.17	
355	1,5-NQ	SO_3H	Full	-196.21	1.18	*
356	1,7-AQ	CHO	R8	-55.05	1.18	
357	1,7-NQ	NO_2	R2	-38.11	1.18	
358	2,3-NQ	PO_3H_2	R4	-80.13	1.18	
359	2,3-AQ	CN	R6	-81.85	1.19	
360	2,3-AQ	PO_3H_2	R5	-97.33	1.19	*
361	2,3-AQ	CN	R5	-76.44	1.19	
362	2,6-NQ	CHO	Full	-70.76	1.19	
363	1,5-NQ	CN	Full	-80.90	1.19	
364	2,3-AQ	COOH	R4	-54.10	1.19	
365	1,2-BQ	CHO	Full	-47.99	1.19	
366	2,3-NQ	SO_3H	R4	-63.15	1.19	
367	1,2-BQ	NO_2	Full	-16.68	1.19	
368	2,3-AQ	CF_3	R10	-32.50	1.20	
369	2,3-AQ	NO_2	R5	-43.60	1.20	
370	1,5-AQ	CN	Full	-109.74	1.20	
371	2,3-AQ	CN	R4	-35.22	1.20	
372	2,3-AQ	COOH	R10	-61.58	1.20	
373	2,3-AQ	CN	R10	-25.50	1.21	
374	1,2-BQ	$COOCH_3$	Full	-46.14	1.21	
375	1,7-NQ	COOH	Full	-163.40	1.21	*
376	2,3-AQ	NO_2	R10	-40.04	1.22	

377	2,3-AQ	CHO	R10	-47.33	1.22	
378	1,2-BQ	PO_3H_2	Full	-168.34	1.23	*
379	2,3-AQ	SO_3H	R10	-64.72	1.23	
380	1,7-NQ	CN	Full	-61.35	1.24	
381	1,7-AQ	NO_2	Full	-27.03	1.25	
382	2,3-AQ	CF_3	R4	-33.79	1.25	
383	1,7-NQ	NO_2	Full	-21.59	1.26	
384	2,6-NQ	CN	Full	-82.25	1.26	
385	2,6-AQ	SO_3H	Full	-152.43	1.27	*
386	2,3-AQ	PO_3H_2	R10	-90.17	1.27	*
387	2,3-NQ	CF_3	Full	-8.59	1.27	
388	2,3-NQ	$COOCH_3$	Full	-61.13	1.28	
389	1,7-AQ	CN	Full	-108.27	1.28	
390	2,3-NQ	$COOCH_3$	R4	-36.72	1.29	
391	2,3-AQ	$COOCH_3$	R4	-39.85	1.29	
392	2,3-NQ	COOH	Full	-152.58	1.30	*
393	2,3-NQ	CHO	R4	-36.05	1.30	
394	2,3-NQ	NO_2	R4	-34.16	1.31	
395	1,5-NQ	NO_2	Full	-21.56	1.32	
396	1,4-BQ	SO_3H	Full	-96.55	1.32	*
397	1,4-BQ	CHO	Full	-46.79	1.34	
398	2,3-AQ	CHO	R4	-43.77	1.36	
399	2,6-NQ	NO_2	Full	-18.68	1.38	
400	2,6-AQ	CHO	Full	-92.83	1.40	*
401	2,3-NQ	CN	Full	-82.23	1.41	
402	2,3-AQ	NO_2	R4	-38.08	1.41	
403	1,7-AQ	CHO	Full	-80.28	1.42	
404	2,6-AQ	CN	Full	-119.30	1.44	
405	2,3-AQ	CN	Full	-100.91	1.49	
406	2,6-AQ	NO_2	Full	-28.48	1.55	
407	2,3-NQ	NO_2	Full	-23.60	1.57	
408	1,5-AQ	SO_3H	Full	-191.41	1.64	*

References for Supplementary Information

1. J. B. Conant, H. M. Kahn, L. F. Fieser and S. S. Kurtz Jr, *J. Am. Chem. Soc.*, 1922, **44**, 1382-1396.
2. J. B. Conant and L. F. Fieser, *J. Am. Chem. Soc.*, 1922, **44**, 2480-2493.
3. V. K. LaMer and L. E. Baker, *J. Am. Chem. Soc.*, 1922, **44**, 1954-1964.
4. J. B. Conant and L. F. Fieser, *J. Am. Chem. Soc.*, 1923, **45**, 2194-2218.
5. J. B. Conant and L. F. Fieser, *J. Am. Chem. Soc.*, 1924, **46**, 1858-1881.
